# Zinc Up-Regulates Insulin Secretion from β Cell-Like Cells Derived from Stem Cells from Human Exfoliated Deciduous Tooth (SHED)

**DOI:** 10.3390/ijms17122092

**Published:** 2016-12-13

**Authors:** Gyuyoup Kim, Ki-Hyuk Shin, Eung-Kwon Pae

**Affiliations:** 1 School of Medicine Department of Obstetrics, Gynecology and Reproductive Sciences, University of Maryland, Baltimore, MD 21201, USA; gykim@fpi.umaryland.edu; 2 UCLA School of Dentistry, Los Angeles, CA 90095, USA; kshin@dentistry.ucla.edu; 3 Department of Orthodontics and Pediatric Dentistry, School of Dentistry, University of Maryland, Baltimore, MD 21201, USA

**Keywords:** dental pulp, insulin, stem cells, zinc, Zrt- and irt-like protein (ZIP)

## Abstract

Stem cells from human exfoliated deciduous tooth (SHED) offer several advantages over other stem cell sources. Using SHED, we examined the roles of zinc and the zinc uptake transporter ZIP8 (Zrt- and irt-like protein 8) while inducing SHED into insulin secreting β cell-like stem cells (i.e., SHED-β cells). We observed that ZIP8 expression increased as SHED differentiated into SHED-β cells, and that zinc supplementation at day 10 increased the levels of most pancreatic β cell markers—particularly Insulin and glucose transporter 2 (GLUT2). We confirmed that SHED-β cells produce insulin successfully. In addition, we note that zinc supplementation significantly increases insulin secretion with a significant elevation of ZIP8 transporters in SHED-β cells. We conclude that SHED can be converted into insulin-secreting β cell-like cells as zinc concentration in the cytosol is elevated. Insulin production by SHED-β cells can be regulated via modulation of zinc concentration in the media as ZIP8 expression in the SHED-β cells increases.

## 1. Introduction

Type-1 diabetes (T1D), a major and difficult-to-manage health problem [1], results from dysfunctional and/or insufficient pancreatic β cells, leading to a loss of insulin as well as glucose homeostasis as the disease advances [2]. Interventions include insulin injection, which isoften accompanied by significant side-effects (e.g.,hypoglycemia), particularly in children. A more amenable approach provides an alternative, supplemental source for the generation of functional insulin-producing β cells using mesenchymal stem cells which can overcome the shortage of donated islets and limitations in the ability to expand β cells in vitro. **S**tem cells from **h**uman **e**xfoliated **d**eciduous tooth (SHED) offer advantages over other stem cell sources for conversion into insulin secreting cells [3,4], including circumvention of ethical issues, noninvasiveness, long-term banking, abundance of tissue source, availability for autologous cells to avoid immunosuppressive regimen for graft rejection responses, and no chance of potential transfection from donors.

Several studies were performed to evaluate “multipotency” and “stemness” of SHED-derived insulin-secreting cell aggregates [5,6,7]; however, few studies focused on what factors control insulin secretion. We previously reported that β cells start losing the capability to secrete insulin before signs of inflammatory cell infiltration or conspicuous cytokine expression emerged in [8], and this notion was supported by such observations in a recent clinical report [9]. We also observed that a lack of zinc results in a significant decline of insulin secretion in conjunction with decreased ZIP8 (**Z**rt- and **i**rt-like **p**rotein 8, a zinc uptake transporter) expression in Sprague-Dawley rats [10]. In accordance with these previous results and other studies [8,10], we presumed that zinc supplementation would up-regulate insulin secretion from insulin-secreting β cell-like cells (or SHED-β cells) via up-regulation of ZIP8. Based on our previous observation and published data, we hypothesized that without zinc accumulation in the cytosol, SHED cannot fully differentiate into insulin-producing SHED-β cells, and therefore, the existance of zinc uptake transporters and maintaining an optimum level of intracellular zinc are critical for maximizing insulin production.

For this preliminary study, we successfully generated SHED-β cells from SHED, based on a published protocol [5]. Using these SHED-β cells, we tested a set of hypotheses: first, if zinc is an essential factor in the regulationprocess of insulin production and secretion. Second, if SHED-β cells contain ZIP8, a zinc uptake transporter. Third, if ZIP8 is involved in the insulin secretion mechanism in SHED-β cells. The purposes of this study were: (1) to demonstrate β cell markers which show SHED differentiating to β cell lineage to become SHED-β cells; (2) to show that ZIP8 expression increasesas SHED-β cell differentiates; (3) to demonstrate the effect of zinc supplementation on insulin secretion.

## 2. Results

### 2.1. SHED Express a Strong “Stemness” Compared to Dental Pulp and Periodontal Ligament Originated Stem Cells (DPSC and PDLSC)

**Figure 1:**
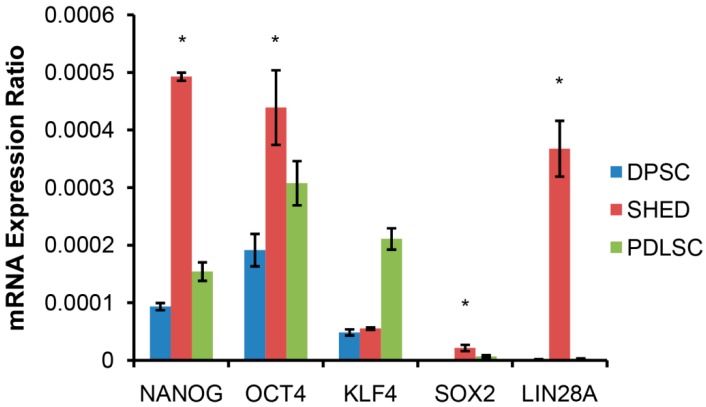
Expression of pluripotent transcription factors determined by quantitative PCR (qPCR) normalized with the level of GAPDH. DPSC: dental pulp stem cells; PDLSC: periodontal ligament stem cells; SHED: stem cells from human exfoliated deciduous tooth. SHED show a significantly stronger expression than other (asterisk * *p* < 0.05) markers, except KLF4. Gene Descriptions: NANOG, Nanog homeobox; OCT4, Octamer-binding protein 4; KLF4, Kruppel like factor 4; SOX2, SRY (sex determining region Y)-box 2; LIN28A, lin-28 homolog A; GAPDH, glyceraldehydes-3-phosphate dehydrogenase.

Results of real-time quantitative PCR (qPCR) assays showed significantly strong expressions of pluripotent stem cell markers in SHED compared to dental pulp stem cells (DPSC) and periodontal ligament originated stem cells (PDLSC) (see Figure 1). This result prompted us to focus on SHED rather than using other stem cell origins. Error bars and P values were calculated from triplicates of each measurements.

### 2.2. Genetic Markers in Pancreatic β Cell Lineage Expressed in SHED Indicate a Successful Conversion of SHED to SHED-β Cells

**Figure 2:**
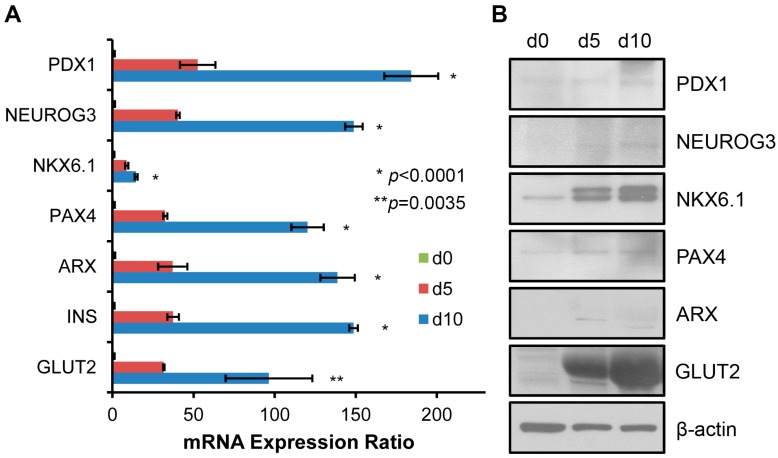
Differentiation of biomarkers in pancreatic lineage shown in the levels of (**A**) mRNA; and (**B**) protein. Every day 5 (or d5) and day 10 (d10) marker expression differed from one another and from d0 in mRNA. *n* = 3. Gene Descriptions: PDX1, pancreatic and duodenal homeobox 1; NEUROG3, neurogenin 3; NKX6.1, NK6 homeobox 1; PAX4, paired box 4; ARX, aristaless related homeobox; INS, insulin; GLUT2, glucose transporter 2.

SHED-β cells differentiated through the three-step media treatment as previously offered by Govindasamy et al. [5] expressed biomarkers in the pancreatic lineage, such as pancreatic and duodenal homeobox 1 (PDX1), neurogenin 3 (NEUROG3), NK6 homeobox 1 (NKX6.1), paired box 4 (Pax4), and aristaless related homeobox (ARX), which were markedly increased between d5 and d10 (see Figure 2).

Particularly, mRNA expressions for insulin (INS) and glucose transporter2 (GLUT2, a main transporter importing glucose molecules into the β-cell cytosol) increased significantly at both mRNA and protein levels. Compared to Day-5 (d5), d10 cells show significantly stronger expression of every gene (see Figure 2A).

### 2.3. ZIP8 Expression and Zinc Supplementation Effects on the Markers in β Cell Lineage

**Figure 3:**
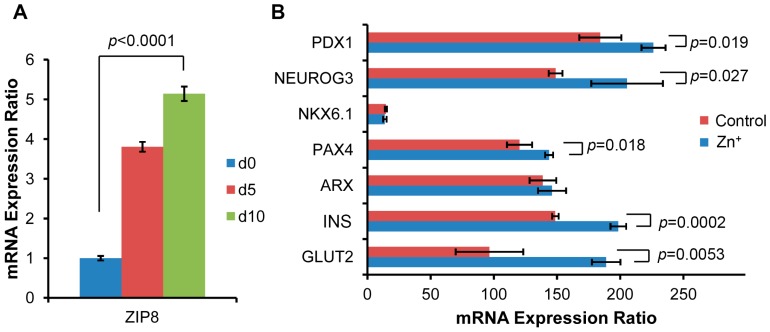
mRNA level changes in Zrt- and irt-like protein 8 (ZIP8) expression and β cell differentiation markers. (**A**) ZIP8 expression increased between d0 and d10; (**B**) Zinc supplementation at d10 increased the levels of most markers, particularly Insulin and GLUT2. *n* = 3.

To examine the hypothesized one-to-one relationship between zinc and the markers expressed in β cell lineage, we measured ZIP8 expression changes because we assumed that the ZIP8 transporter mediates the insulin production and secretion in SHED-β cells (Figure 3). The transcriptional activity of the ZIP8 gene increased significantly as differentiation proceeded (See Figure 3A).

Zinc (50 µM) was added to the media on day 10. This supplementation of zinc increased not only the genes in the β cell lineage, but also INS and GLUT2. This may indicate that zinc supplementation could augment insulin secretion.

### 2.4. Levels of Zinc in Conjunction with ZIP8 in the Cytosol of SHED-β Cells Modulate Insulin Secretion

**Figure 4:**
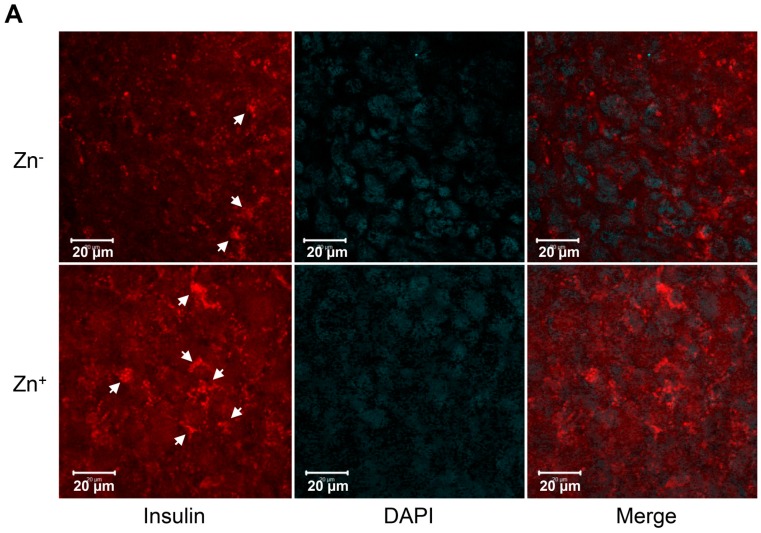

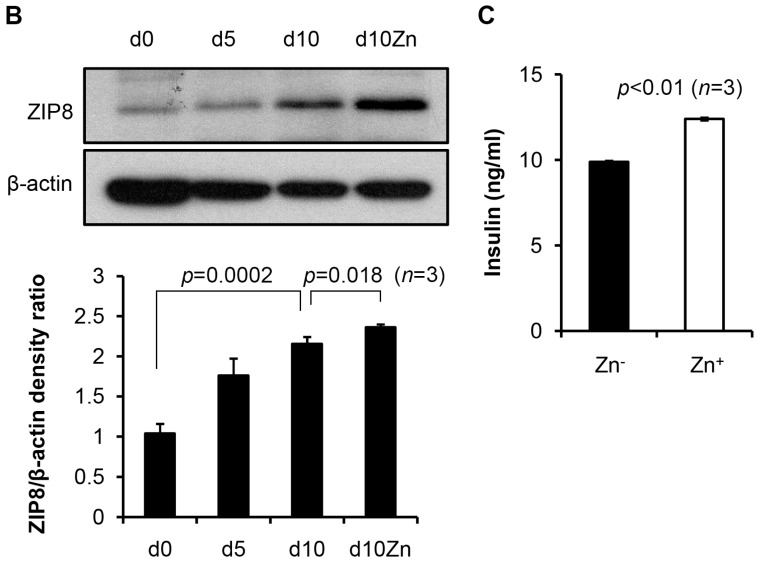
Zn supplementation affects the production and secretion of insulin and the expression of ZIP8 in SHED-β cells. (**A**) Immunofluorescence staining of insulin (indicated by red color and white arrow heads) in SHED-β cells with or without zinc supplementation. Note the more prominent expression of insulin after zinc supplementation in the merged panel; (**B**) Western blot analysis of ZIP8 expression in different time points with or without zinc supplementation; (**C**) Constitutive secretion of insulin from SHED-β cells evaluated by ELISA on *n* = 3. In these assays, 50 µM of zinc was supplemented.

To investigate the roles of zinc in the cytosol of SHED-β cells in association with ZIP8 expression, we augmented the zinc concentration of the media by 50 µM, and then we compared before and after supplementation. Figure 4 contrasts the changes in SHED-β cells in the images obtained by immunofluorescence staining. Figure 4A demonstrates a marked increase in the number of insulin-containing β cell-like stem cells after zinc supplementation. Insulin molecules in the cytosolare shown in brighter red color after the augmentation (Figure 4A). Figure 4B demonstrates a significant increase of ZIP8 protein after zinc supplementation. Lastly, supplementation of 50 µM of zinc significantly increased insulin secretion by approximately 25% (9.8 ± 0.025 vs. 12.39 ± 0.035 ng/mL) compared to control media, as shown in Figure 4C).

## 3. Discussion

Several previous studies thoroughly demonstrated the process by which mesenchymal stem cells from various sources could convert to insulin-secreting cells [3,4,6]. However, most studies focused on proving the pluripotency of their cells or showing β-cell-like phenotypes of their cells. A scarce number of studies have reported what factors play a key role in influencing quantity and quality of insulin secretion. Based on previous experience and understanding, our study focused on what factors play a key role in the augmentation of insulin secretion. The results of this study support a new concept that zinc and zinc uptake transporters (particularly ZIP8) may affect the proliferation of SHED-β cells. Zinc is an essential element involved in many basic cellular biochemical processes for human physiology. Zinc plays its roles as a part of protein structure, signaling process, and enzymatic regulation [11,12]. For instance, when zinc and osteocalcin are lacking during perinatal growth, brittle bones in rats [13] and a short height [14] may result. Conversely, Zinc supplementation dampens osteoclastic activity [15] and upregulates osteoprotegerin [16] in diabetic rodents and cell lines. Zinc in calcium phosphate is known to modulate bone induction [17] and mineralization [18]; therefore, zinc plays a significant role in bone formation. Bones in patients with type 1 diabetes often suffer from a lack of bone (i.e., osteopenia and osteoporosis) [19,20,21]. Indeed, a lack of bone in diabetes is associated with zinc deficiency [22,23].

Zinc homeostasis in the cytosol is tightly regulated via ubiquitous zinc-bound intracellular proteins such as metallothionein as well as zinc pool in intracellular organelles such as Golgi apparatus. Due to these reservoirs inside the cells, pathologic symptoms of zinc depletion cannot be easily recognized [24]. Particularly, insulin-producing pancreatic β cells are specified to contain a high level of zinc because insulin hexamer molecules are structured around zinc ions [25]. In addition, β cells are exposed to oxidative stress due to reactive oxygen species (ROS) produced during the cleaving process from proinsulin to insulin and during glucose metabolism, which consume anti-oxidants such as zinc containing metallothionein [26,27]. Thus, zinc is an essential metal for synthesizing insulin.

Previous studies have shown that lack of zinc in pancreatic islets is associated with reduced insulin secretion [8,10]. Our current results indicate that zinc supplementation assists the process of converting SHED into SHED-β cells. In addition, this augmentation is strongly associated with the increased expressions of ZIP8 in SHED-β cells. A recent publication revealed that their culture media contained 10 µM Zn^2+^ [4]. Then, since we used 50 µM, a question would merge if 10 µM of zinc is optimal.

Zinc transporters are cell structures involved in regulating zinc homeostasis. Therefore, questions must start from which zinc transporter(s) play a role in zinc homeostasis. We reported that one of the zinc uptake transporters—Zip8—plays a major role in insulin secretion [10] using primary β cells harvested from rats. More recently, a research group investigated a role of Zip4 in insulin secretion; however, Zip4 zinc uptake transporters were not directly associated with insulin secretion in mice [28]. Another independent study concluded that ZIP6 and ZIP7 are functionally important [29] for maintaining zinc homeostasis in human islets and β cell lines; however, they did not measure changes in insulin secretion in pancreatic cells in response to ZIP expression changes. Therefore, ZIP8 may be an unknown zinc uptake transporter involved in the regulation of insulin production and secretion. We confirmed that ZIP8 may be an important functional transporter in SHED-β cells essential for optimal production of insulin via the regulation of intracellular zinc levels. We found that ZIP8 up-regulation for increased accumulation of zinc is an important factor in the differentiation process of SHED into SHED-β cells.

Finally, our findings could develop an ex vivo study model determining what important factors play a major role as zinc and ZIP8 augmented SHED-β cells are transplanted to human β cells aggregates. Glucose-stimulated insulin secretion studies on SHED-β cells with and without zinc supplementation would confirm our conclusion. Future experiments to determine the optimized conditions for the mechanism involved in zinc regulation for the optimal insulin production in β cells is a critical point for understanding clinical implications in not only Type-1 but Type-2 diabetes mellitus [30].

## 4. Methods

### 4.1. Cell Cultures

Three types of human dental mesenchymal stem cells were cultured and used for the study. Primary SHED cells were isolated from normal exfoliated deciduous incisors. Dental pulp stem cells (DPSCs) were isolated from extracted teeth. Periodontal ligament stem cells (PDLSCs) were scraped from harvested third molars. All three cell types were cultured in α-minimum essential media (or α-MEM)medium (Invitrogen, Carlsbad, CA, USA) supplemented with 10% fetal bovine serum (Invitrogen), 5 μg/mL gentamicin sulfate (Gemini Bio-Products, West Sacramento, CA, USA), and 20 mmol/L l-glutamine (Invitrogen).

### 4.2. In Vitro SHED-β Cell Differentiation

Collected SHED cells were amplified to passage 5 in the media, as described above. SHED-β cells were differentiated using the previously established protocol with slight modifications [5]. After trypsin-treated and centrifuged, the cells were resuspended in Medium-A (containing Dulbecco’s modified Eagle’s medium/F-12 Knock-out (DMEM-KO), 17.5 mM Glucose, 1% bovine serum albumin Cohn Fraction V (BSA-CF; fatty acid free), 1× insulin-transferrin selenium (ITS), 4 nM Activin A, 1 mM Sodium Butyrate, 50 µM 2-Mercaptoethanol, and 5 µg/mL gentamicin sulfate) and plated in sterilized borosilicate glass plates (60 mm, Corning, Keene, NH, USA) without serum. After the cultures were incubated for 48 h, the medium was changed to Medium-B (containing DMEM-KO, 17.5 mM Glucose, 1% BSA-CF, 1× ITS, 0.3 mM taurine, and 5 µg/mL gentamicin sulfate), and was substituted with Medium-C (DMEM-KO, 17.5 mM Glucose, 1.5% BSA-CF, 1× ITS, 3 mM Taurine, 100 nM glucagon-like peptide-1, 1× non-essential amino acids, and 5 µg/mL gentamicin sulfate) on the fifth day. Gravity-downed aggregated cell pellets were gently aspirated, and the medium was replaced with fresh Medium-C every 2 days for the next 5 days. To make the zinc-rich environment for the differentiation step, 50 µM zinc chloride was added in every differentiation medium. All reagents were purchased from Sigma-Aldrich, St. Louis, MO, USA; Invitrogen, Carlsbad, CA, USA; R&D Systems, Minneapolis, MN, USA. 

### 4.3. Real-Time qPCR

Stem cells and differentiated SHED-β cells were harvested, and total RNAs were purified using RNeasy Mini Kit (QIAGEN Sciences, Germantown, MD, USA) in accordance with the manufacturer’s protocol. First-strand cDNA was synthesized from 1 µg of RNAs using the High Capacity cDNA Reverse Transcription Kits (Applied Biosystems, Foster City, CA, USA) primed with a mixture of random primers. A 2 µL volume of cDNA template was used on the mixture of 25 µL volume of SYBR Green master mix (Applied Biosystems, Carlsbad, CA, USA) with 5 pmol of primers. The primer sequences (5 to 3 primes) for each gene are the following:

NANOG, F: AGATGCCTCACACGGAGACT; R: TCTCTGCAGAAGTGGGTTGTT.
OCT4, F: GAAAACCCACACTGCAGATCA; R: CGGTTACAGAACCACACTCG.
KLF4, F: GGGAGAAGACACTGCGTCA; R: GGAAGCACTGGGGGAAGT.
SOX2, F: TCTCATGATGTTCAACCATTCAC; R: CACATTTACATTCAAAGCACCAG.
LIN28A, F: GAAGCGCAGATCAAAAGGAG; R: GCTGATGCTCTGGCAGAAGT.
GAPDH, F: GGTGTGAACCATGAGAAGTATGA; R: GAGTCCTTCCACGATACCAAAG.
PDX1, F: GGGTGACCACTAAACCAAAGA; R: GGTCATACTGGCTCGTGAATAG.
NEUROG3, F: GCTGCTCATCGCTCTCTATTC; R: GGCAGGTCACTTCGTCTTC.
NKX6.1, F: GAAGAGGACGACGACTACAATAAG; R: CTGCTGGACTTGTGCTTCT.
PAX4, F: TGGGAAGGAGATGGCATAGA; R: ATCACAGGAAGGAGGAAGGA.
ARX, F: GGCAAGGAGGTGTGCTAAA; R: GCTGGTCCTCTGTTTCCATT.
INS, F: CTGGAGAACTACTGCAACTAGAC; R: TGCTGGTTCAAGGGCTTTAT.
GLUT2, F: CCGCTGAGAAGATTAGACTTGG; R: GACTAGCTCCTGCCTGTTTATT.
SLC39A8, F: GCTGGCTATTGGGACTCTTT; R: GCAACTGCCTTCTCAACATAAC.
ACTB, F: GGATCAGCAAGCAGGAGTATG; R: AGAAAGGGTGTAACGCAACTAA.

Quantitative PCR reactions were triplicated for each sample with the Eppendorf Realplex System (Eppendorf, Hamburg, Germany), and the threshold cycle (*C*_t_) for each reaction was normalized (Δ*C*_t_) by the value of the β-ACTIN (ACTB) housekeeping gene. The value of Δ*C*_T_ was further normalized to exhibit the comparative expression levels with respect to the mean value.

### 4.4. Western-Blots

Collected SHED and SHED-β cells were lysed in ice-cold RIPA buffer containing protease inhibitor cocktails (Roche Applied Science, Indianapolis, IN, USA). The same amount of proteins was resolved on the SDS-PAGE and transferred onto a polyvinylidene fluoride (PVDF) membrane using an electroblot. After blocking with 5% milk TBS-T, the membrane was incubated with anti-PDX1, anti-β-actin (Cell Signaling Technology Inc., Beverly, MA, USA), anti-NEUROG3, anti-ARX, anti-GLUT2 (Sigma-Aldrich, St. Louis, MO, USA), anti-NKX6.1 (R&D Systems, Minneapolis, MN, USA), anti-PAX4 (GeneTex Inc., Irvine, CA, USA), and anti-ZIP8 (Thermo Scientific Inc., Rockford, IL, USA) antibodies. A horseradish peroxidase-conjugated secondary antibody was added, and chemiluminescent reagents were used to detect immunoreactive proteins on X-ray films. A density measurement was performed on Multi Gauge V3.0 (Fujifilm, Tokyo, Japan), and the quantities were calculated by subtraction between ZIP8 and β-actin bands.

### 4.5. Immunofluorescence Assay

On the ninth day of differentiation, aggregated SHED-β cells were transferred on the Lab-Tek II CC2 Chamber Slide (Thermo Scientific Inc., New York, NY, USA) and incubated for 24 h to allow proper attachment on the surface. The cells were fixed with CytoCell Fixative solution (Biocare Medical, Concord, CA, USA) for 20 min, and incubated in blocking solution (CAS-BLOCK; Invitrogen, Halethorpe, MD, USA) for 15 minutes at room temperature. SHED-β cells were stained with anti-Insulin (GenScript, Piscataway, NJ, USA) antibody at room temperature for 2 hours, washed three times with PBS, and then incubated with Alexa Fluor 594-conjugated secondary antibody (Invitrogen, Eugene, OR, USA) for 1 h. After being washed with PBS, the slide was mounted in Vectashield mounting medium containing DAPI (4′,6-diamidino-2-phenylindole) (Vector Laboratories, Burlingame, CA, USA). Fluorescent images of the samples were obtained using a Zeiss LSM530 META Confocal Microscope (Carl Zeiss, Thornwood, NY, USA).

### 4.6. ELISA Assay for Insulin

On day 10 of the differentiation of SHED-β cells, incubation medium was refreshed and maintained for 2 h to allow insulin secretion. The media were collected to quantify insulin levels using Insulin ELISA Kit (EMD Millipore Corp., Billerica, MA, USA). An equal amount of sample was incubated on the anti-insulin antibody-coated plate with biotinylated capture antibody for 90 min. After the plate was washed, a horseradish peroxidase-conjugated streptavidin was added, and TMB (3,3′,5,5′-tetramethylbenzidine) substrate and stop solution were mixed for color developing reaction. Absorbance was measured at 450 nm using a spectrophotometer (BioTek Instrument Inc., Winooski, VT, USA).

### 4.7. Statistical Analysis

For group comparison, Student’s *t*-tests were performed with SPSS v. 21 (IBM Co., Armonk, NY, USA). Significance level of *p* < 0.05 was adopted for inference tests on mRNA and protein measurements. Samples collected from multiple teeth were used for triplicated measurements.

## 5. Conclusions

(1) β cell-differentiation markers emerge when SHED convert into SHED-β cells; (2) ZIP8 expression increases as SHED differentiate into SHED-β cells; (3) zinc supplementation enhances insulin secretion by SHED-β cells; (4) insulin secretion is augmented by ZIP8 elevation.
